# The significance fallacy in inferential statistics

**DOI:** 10.1186/s13104-015-1020-4

**Published:** 2015-03-17

**Authors:** Anton Kühberger, Astrid Fritz, Eva Lermer, Thomas Scherndl

**Affiliations:** Department of Psychology and Centre of Cognitive Neuroscience, University of Salzburg, Hellbrunnerstr. 34, 5020 Salzburg, Austria; Österreichisches Zentrum für Begabtenförderung und Begabungsforschung, Salzburg, Austria; Department of Psychology, University of Munich, Munich, Germany

**Keywords:** Statistical significance, Practical significance, Effect size, NHST, Sample size

## Abstract

**Background:**

Statistical significance is an important concept in empirical science. However the meaning of the term varies widely. We investigate into the intuitive understanding of the notion of significance.

**Methods:**

We described the results of two different experiments published in a major psychological journal to a sample of students of psychology, labeling the findings as ‘significant’ versus ‘non-significant.’ Participants were asked to estimate the effect sizes and sample sizes of the original studies.

**Results:**

Labeling the results of a study as significant was associated with estimations of a big effect, but was largely unrelated to sample size. Similarly, non-significant results were estimated as near zero in effect size.

**Conclusions:**

After considerable training in statistics, students largely equate statistical significance with medium to large effect sizes, rather than with large sample sizes. The data show that students assume that statistical significance is due to real effects, rather than to ‘statistical tricks’ (e.g., increasing sample size).

## Background

There is continuing debate on the usefulness and validity of the method of Null Hypothesis Significance Testing (NHST, e.g., [1-3]). Several journals edited special issues on this topic (e.g., *Journal of Experimental Education* in 1993; *Psychological Science* in 1997; *Research in the Schools* in 1998) that culminated in the question: What is beyond the significance test ritual (*Journal of Psychology* in 2009)?

The debate has led to an increased awareness of the problems associated with NHST, and these problems are linked to what has been referred to as a ‘crisis of confidence’ [4]. Among the dominant recommendations for NHST is reporting of effect size as a supplement to the *p* value [5]. Accordingly, not only the statistical significance of a result should be valued but also the effect size of the study (e.g., [1,6-12]). This should prevent readers from holding the false belief that significant results are automatically big and important, or otherwise, that not significant means ‘no effect at all’. Although these misconceptions, that significance means big, and non-significance means no effect, are often referred to in the literature (e.g., [3,13-17]) their empirical basis is weak. This is clearly in conflict with the demand for evidence based practice in statistics and statistics education [18]. Thus, the purpose of the present study was to investigate the prevalence of these misconceptions.

### Statistical and practical significance

The distinction between statistical and practical significance is quite old. The origin of statistical significance can be traced back to the 1700s [19]. Practical significance, expressed as the strength of the relationship between two variables, can roughly be dated back to the 18th century [20]. Modern statistical significance refers to the *p* value as the result of a significance test. If p < .05 a result is statistically significant. This notion of statistical significance became popular in the social sciences in the first half of the 20th century mainly due to the work of Sir Ronald Fisher [21,22]. With the rise of the statistical significance test, the concept of effect magnitude became seemingly dispensable. Only recently, there is an opposite trend and many authors pointed to the importance of reporting the magnitude of the effect under investigation, mostly because statistical tests are so heavily influenced by sample size (e.g. [6,23-32]). Recall that a test statistic is the product of sample size and effect size [16,33]. The *p* value, as a common-language translation of the various test statistics [8], is therefore also a function of practical significance and sample size, in short: *p = f (ES, N)*. If the effect is small but the sample size very large, the *p* value will be statistically significant. Similarly, if the effect size is large and the sample size small, the *p* value will also be significant. Thus, given a big enough sample, even trivial effects can be statistically significant [34]. A correct interpretation of the significance test therefore requires taking the relationship between sample size and effect size into account [35].

Consider the classic aspirin textbook example in Rosnow and Rosenthal [36]: A study tested the effect of aspirin on reducing heart attacks. 11.034 men were given an aspirin pill to be taken every 2 days, whereas 11.037 other men were given a placebo. Statistically speaking, the treatment was enormously effective (p < .000001). It was so effective that it was decided to end the study prematurely because the outcome was clear and it appeared unethical to deprive the participants of the control group of the beneficial aspirin [37]. Here statistical significance was equated with practical significance. However, the treatment was far from being effective in terms of effect size (*r*^2^ = .0011): statistical and practical significance can tell different stories.

The failure to distinguish between statistical and practical significance has been called the *significance fallacy* [17]. It comes in two varieties. The first variety is to equate a low *p* value with a big effect size. Thus, the numeric value of *p* is considered as an indicator of the strength of the treatment effect under test, or the distance between the groups that are compared. Kline [16] called this the *magnitude fallacy*. The second variety of not distinguishing between statistical and practical significance is that statistically non-significant results are interpreted as evidence of no effect, as ‘no difference between means’, or as ‘no relationship between variables’. We call this the *nullification fallacy.*

The nullification fallacy has the potential to damage science (and lives), as an example taken from Fidler (Fidler F: From statistical significance to effect size estimation: statistical reform in psychology, medicine and ecology. Unpublished PhD thesis, University of Melbourne, 2006) shows: in ecology, mark-and-recapture studies are used to determine population sizes. To identify individual frogs upon recapture researchers used to clip certain combinations of toes in order to spare the sensitive skin of the animals, since some studies investigating whether toe-clipping had an impact on the frog’s survival rates found no significant effects. But when Parris and McCarthy [38] reanalysed the evidence they found that toe-clipping did actually decrease the survival rate by 6–18% with each toe clipped. The sample sizes of the original studies were just too low to (statistically) show the effect. In this example, the consequence of misinterpreting a non-significant result as indicating that there is no effect is obvious. Other researchers have heatedly argued about the negative consequences of uncritically using underpowered studies to declare the null hypothesis true (for animal studies see [39]; for Neuroscience, see [40]).

### Previous research

It is difficult to estimate how common these fallacies about statistical and practical significance are, although these misconceptions are discussed in virtually every article reviewing NHST (e.g. [3,13-17]). Among the first experimenters to examine the level of p < .05 were Rosenthal and Gaito [41,42], who found an abrupt drop in confidence in a *p* level just above .05. Nelson et al. [43] later found this *cliff effect* in a survey on psychological researchers. Again and again the magical nature of p < .05 has been shown (e.g., [44]), with some authors proposing strategies for adjusting effect size estimates taking the publication bias into account (e.g., [45,46]). More recent research investigated the consequences of the cliff effect with an eye on the unhealthy effect of small (i.e., underpowered) - however significant – studies for psychological research in general (e.g., [47,48]).

**Figure 1:**
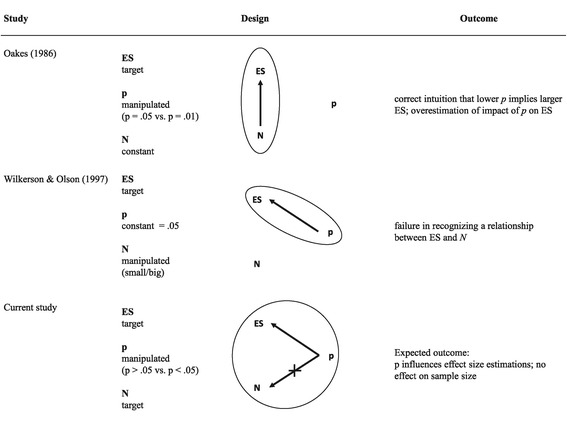
**Overview of previous studies investigating the understanding of the relationship between effect size (ES), and sample size (N).**

Among the range of studies discussing the consequences of NHST, two empirical studies investigated intuitions about the relationship between effect size, sample size and *p*. Oakes [49] asked academic psychologists to estimate the unstandardized effect size for a given Student’s *t*-test example. Oakes prescribed the *p* value and sample size and had participants estimate the size of the effect (see Figure 1). He found that the effect size was generally overestimated. In addition, for identical sample sizes, participants understood that the effect size associated with a *p* value of .01 is bigger than the effect size associated with a *p* value of .05. The increase in effect size for *p* = .01 is normatively correct, but participants overstated it considerably. That is, as the *p* decreased the effect size was assumed to increase disproportionally. This can be seen as an instance of the *magnitude fallacy.*

Wilkerson and Olson [35] investigated how graduate students understand the relationship between effect size, sample size, and errors of statistical inference by asking which of two studies, one with a small and one with a big sample size, provides better evidence when both are statistically significant at *p* = .05. Only one out of 52 graduate students recognized that, given two different studies reporting the same *p* value, the study with the smaller sample size indicates the larger effect. Missing the link between sample size and effect size is also indicated in the legendary hospital problem by Kahneman and Tversky [50], where participants failed to recognize that a smaller hospital is more likely to have an uneven portion of male vs. female babies born than a large hospital.

In Figure 1 the studies of Oakes [49] and Wilkerson and Olson [35] are schematically depicted, and the main outcomes are reported. As can be seen, each study focused on a specific part of the relationship between effect size, sample size and *p* (circled in Figure 1), holding one parameter constant. Here we extended this design to include all three variables concurrently for understanding the relationship between effect size, sample size and *p* inside the heads of our participants.

### The present research

In the present research we examined whether students associate statistical significance with practical significance, as stated by the *significance fallacy*. We did this by testing both aspects of the *significance fallacy*: the *magnitude fallacy* and the *nullification fallacy*. According to the *magnitude fallacy* statistically significant results will be rated as having a higher effect size than non-significant results; according to the *nullification fallacy,* non-significant results will be rated as zero in effect size. In essence, we investigated intuitions about effect sizes and sample sizes in the context of *p*-values: is a significant *p* value due to effect size or due to sample size?

Our procedure was as follows: we picked two published studies and described the procedure and the aim of these studies, including the main hypothesis and the dependent measure. We also described whether or not the finding was statistically significant. Note that we did not report statistical measures such as sample size, means, or standard deviations; we rather had participants estimate these measures. That is, we reported the interpretation of the findings and had participants estimate the data. Statistical inference goes usually the other direction, from data to interpretation. In order to investigate intuitions about data, reversing the inferential direction can lead to important insights. In particular, we compared participants’ estimations in the significant vs. the non-significant condition. In this way we were able to test whether people expect the difference between statistical significant and non-significant result in the effect size, the sample size, or both.

## Methods

### Participants and procedure

We sampled 214 students of psychology (156 females, mean age = 23.5, SD = 6.81) from the University of Salzburg enrolled in a statistics course as participants. Sampling was done in different years, thus the sample is of different cohorts whose statistics education is similar, however. All participants were familiar with hypothesis testing and with the concept of statistical inference due to three previous statistics courses (each 3 hours/week). Students participated during their regularly scheduled class time. To ensure commitment participants were offered the chance of winning 20 Euro. The prize was awarded to the two students who came closest to the actual sample size. Under Austrian law it is not necessary to seek formal ethical approval for conducting this research.

### Material and design

We selected two published studies from *Psychological Science:* ‘Thermometer of social relations’ [51] and ‘Body locomotion as regulatory process’ Koch et al. [52]. The ‘thermometer’-study investigated the influence of different temperatures on social relations. It was tested whether participants rated their social proximity to another person as closer when holding a warm compared to a cold beverage. Proximity was measured on a scale ranging from 1 to 7. The ‘locomotion’-study addressed the significance of the motor system in influencing cognitive processes. It tested whether stepping backwards enhances cognitive control, measured by a Stroop test, in comparison to stepping forward. Reaction time in ms was the outcome variable of interest. These two articles were chosen because their main research question and their main outcome variable are easily comprehensible. Both studies used t-tests for the analysis.

Participants were presented a short description of each study of approximately 150 words including research question, method, design (i.e. between groups), and outcome variable (see Appendix) Subsequently, participants were asked to estimate the respective measures as they would expect them to be reported in the results section of the paper (i.e., sample sizes, means and standard deviations of both groups, Cohen’s d [53]). All participants were presented both studies consecutively, whereas one was described as statistically significant and the other as non-significant. Note that therefore participants did not rate both, the significant and the non-significant condition of the same study. The sequence of the studies was altered between groups. From the participants’ ratings we computed an unstandardized effect size (mean difference, calculated by subtracting the means of the two groups).

## Results

An initial survey of the data indicated that some of our participants were unable or unwilling to follow the instruction. To ensure adequate data quality we therefore settled for a rigorous regime of data inclusion. In a first step, in the thermometer-study 13 participants had to be excluded because estimates were beyond the range of the response scale. We then excluded participants with missing values in our main dependent variables (n_thermo_ = 6; n_locomotion_ = 10), and participants showing signs of inconsistency between *p* level and condition, either by giving *p* values larger than 1 (n_thermo_ = 16; n_locomotion_ = 20), by reporting significant *p* values in the non-significant condition (n_thermo_ = 25; n_locomotion_ = 23), or by providing non-significant *p* values (p > .05) in the significant condition (n_thermo_ = 28; n_locomotion_ = 28). This led to a final sample size of 127 participants in the thermometer scenario, and 133 participants in the locomotion scenario. In terms of power, we achieved a power larger than 0.80 to detect a difference between conditions of d = .50, p < .05, one-sided test, in both scenarios.

The assumption of normal distribution was violated for the mean difference ratings and for the sample size ratings. In addition, several outliers were included in our data. We therefore computed non-parametric analyses (Mann–Whitney U-Tests) to assess differences between the two conditions. The ratings of sample sizes, median values, and standard deviations, as well as the resultant unstandardized and standardized effect sizes are presented in Tables 1 and 2 for the ‘thermometer’ and ‘locomotion’-study, respectively. For every variable three values are given: (i) the actual result as reported in *Psychological Science*, (ii) the estimations of the participants in the significant condition, and (iii) the estimations of the participants in the non-significant condition. Due to non-normality, we report medians for the two latter conditions. We found neither effects of order of the scenarios (z <-1.01, p > .30, r < .09) nor of the order of significant and non-significant condition (z <-1.19, p > .23, r < −.10) on our dependent variables and thus collapsed these conditions in the further analysis.

**Table 1:** **Results for ‘thermometer’-study**

	**Actual** ^**a**^	**‘Significant’ (n = 53)**	**‘Non-significant’ (n = 73)**	**(z-value)** ***p-value***	**Effect size**
N	33	76	50	(z =-1.75) *p* = .08	r = -.15
M _group1_	5.12	2.70	3.50		
M _group2_	4.13	4.05	4.00		
M_diff_	0.99	2.00	1.00	(z =-5.27) *p* < .001	r = -.47
SD _group1_	1.22	1.00	1.25		
SD _group2_	1.41	8.00	10.00		
Cohen’s *d*	0.78^b^	0.60	0.30	(z =-3.88) *p* < .001	r = -.34

Note. ^a^The actual study reported a significant effect. Attempts to replicate the effect of temperature on social relations within the Many Labs Replication Project have failed, however [54].

^b^Cohen’s d = .78 is reported in the paper. Calculating effect size from means and standard deviations using the Campbell effect size calculator available at http://www.campbellcollaboration.org/resources/effect_size_input.php results in d = .75, 95% C.I = [0.05; 1.46].

**Table 2:** **Results for ‘locomotion’-study**

	**Actual** ^**a**^	**‘Significant’ (n = 65)**	**‘Non-significant’ (n = 68)**	**(z-value)** ***p-value***	**Effect size**
N	38	60	50	(z =-0.90) *p* = .37	r = -.08
M _group1_	712	150	150		
M _group2_	676	120	118		
M_diff_	36	50	10	(z =-2.48) *p* = .013	r = -.21
SD _group1_	83	10	5		
SD _group2_	95	8	5		
Cohen’s *d*	0.79	0.70	0.20	(z =-4.16) *p* < .001	r = -.36

Note: ^a^ The actual study reported a significant effect.

### Testing the magnitude fallacy

According to the magnitude fallacy*,* statistically significant results will be rated as having a higher effect size than non-significant results. Therefore we tested whether the absolute unstandardized (M_diff_), and the standardized effect size (Cohen’s *d*), were higher in the significant than in the non-significant condition. We found that participants in the significant condition estimated both effect size measures (M_diff_ and Cohen’s *d*) higher than participants in the non-significant condition. These findings were consistent in both studies (‘thermometer’-study, for M_diff_: z =-5.27, p < .001, r = −.47; for d: z =-3.88, p < .001, r = −.34; c.f. Table 1; ‘locomotion’-study, for M_diff_: z =-2.48, p = .013, r = −.21; for d: z =-4.16, p < .001, r = −.36; c.f. Table 2). Note also that the effect size estimates in the significant conditions were quite close to the actual data reported in *Psychological Science.*

Inspection of Tables 1 and 2 shows that participants rated the sample sizes in the significant conditions only slightly larger than in the non-significant conditions. These effects were small (r = −.15, and r = −.08, respectively), and statistically non-significant in both studies. Interestingly, the estimated sample sizes were consistently higher than the sample size of the actual study, which was quite low, however.

Another way to present the results is in terms of ratios of the effect sizes and sample sizes between the significant and non-significant condition of each study. The effect sizes were in both studies rated higher in the significant compared to the non-significant condition (M_diff_: 2 : 1 and 5 : 1; Cohen’s *d*: 2 : 1 and 3.5 : 1, for locomotion and thermometer study, respectively). In contrast, sample sizes were rated only slightly higher in the significant condition in the ‘thermometer’-study (1.5: 1) and similarly in the ‘locomotion’-study (1.2 : 1).

### Testing the nullification fallacy

**Figure 2:**
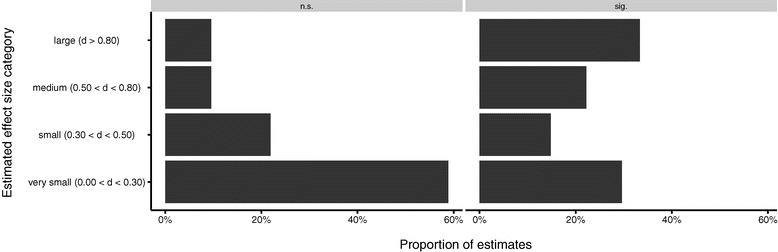
**Proportions of participants estimating the effect as very small (0.00 < d < 0.30), small (0.30 < d < 0.50), medium (0.50 < d < 0.80), or large (d > 0.80), for both studies.**

According to the *nullification fallacy* statistically non-significant findings will be interpreted as evidence of no effect and therefore the effect size should be rated as approximately zero. We found that only a minority of our participants specified the mean difference or Cohen’s d as exactly zero (6% in ‘thermometer’- study, and 3% in locomotion study, respectively). However, a large proportion of participants thought that an effect in a non-significant study is very small (59% in the ‘thermometer’-study, and 60% in the ‘locomotion’-study; cf. Figure 2). Table 3 shows the exact distribution in terms of d values: as can be seen, non-significant studies were considered to have very small or small effects, whereas significant studies were estimated as more diverse: very small as well as large effects were estimated. In sum, although most participants did not specify the difference between the two means, or Cohen’s d, as exactly zero, they guessed that statistically non-significant findings might also be of low practical significance.

**Table 3:** **Crosstabulation of estimated Cohen’s d for significant and non-significant condition for both studies**

**Category**	**Thermometer study**		**Locomotion study**	
	**‘Significant’ (n = 53)**	**‘Non-significant’ (n = 73)**	**‘Significant’ (n = 65)**	**‘Non-significant’ (n = 68)**
Large (d > 0.80)	17 (30%)	7 (9%)	24 (37%)	11 (16%)
Medium (0.50 < d < 0.80)	12 (15%)	7 (9%)	19 (29%)	4 (6%)
Small (0.30 < d < 0.50)	8 (23%)	16 (22%)	13 (20%)	12 (18%)
Very small (0.00 < d < 0.30)	16 (30%)	43 (59%)	9 (13%)	41 (60%)

## Discussion

This study tested two fallacies associated with statistical significance: the *magnitude fallacy* and the *nullification fallacy*. According to the *magnitude fallacy* results accompanied by low *p* values are interpreted as having a higher effect size than results with higher *p* values. Effects of non-significant results will, according to the *nullification fallacy,* be interpreted as evidence of no or a negligible effect.

We found that significant results were rated to have higher effect sizes compared to non-significant results. In contrast, sample sizes were not rated higher in the significant condition. That is, in the formula *p = f (ES, N)* only the effect size seems to be considered, but not the sample size. This could indicate that students assumed that the presented studies have used power analysis to attain the adequate sample size for their experiments. In power analysis the relationship between effect size and samples size is optimized: sample sizes are chosen to be ‘big enough’ so that an effect of such magnitude as to be of scientific significance will also be statistically significant, but sample sizes will not be ’too big’, so that an effect of little scientific importance is not statistically detectable [55,56]. However, power surveys of psychological articles reveal again and again (e.g., [57-63]) that the probability of finding a significant effect of medium effect size (i.e., *r* = .30, *d* = .50) is in the range of 0.40–0.60. This implies that the ‘optimal’ sample size is rarely calculated. Note also that power analysis is virtually never reported in journal articles as has been shown in a current review [64] that assessed and reanalysed reporting practices of over 6000 educational and psychological articles. This neglect of power is not surprising given that the concept of power arose out of the Neyman-Pearson approach of hypotheses testing which seems to be very seldom used [65].

However, even if researchers (and teachers) fail in using power considerations in their research, it could still be that students rely on power calculations, since this is what they (at least our students) are told in their research methods class. Frequently power considerations have been part of the curricula in the last years, but these ideas seem to meet with little love in current research. In any case, we do not think that considerations of power are the basis for expecting large effect sizes for significant findings.

It is one important feature of our findings that students estimated effect sizes to be different for significant and non-significant findings. Notice that the significant condition was a description of the original study. Thus we can see how close our participants’ estimates came to the actual findings. In terms of Cohen’s d, participants did not overestimate the effect size, not even in the significant condition. Note, however, that the actual finding of d = 0.75 in the thermometer study is a very imprecise estimate, with the 95% C.I. for d ranging from d = 0.05 to d = 1.46. Hardly any plausible positive effect size estimate can be far off this value. For the locomotion study this presumably also applies, but it is impossible to calculate the exact 95% C.I. from the data due to the within subjects design and the failure to report the correlation between the conditions.

We found only partial support for the nullification fallacy, that non-significant effects will be actually rated as zero in effect size. Only few participants rated the effect in the non-significant condition as exactly zero in size. Practically, this expectation is highly unlikely in the first place as students know that there is actually always a difference in some decimal place between two sample means (cf. the fallacy of soft psychology, [31]). However, although the majority of participants did not predict an effect size of exactly zero, they predicted a lion’s share of negligible to small effects in the non-significant condition. A comparison of estimated effect sizes in the significant condition shows a striking difference: here mainly medium to large effect sizes were predicted. People thus do not nullify, they rather minimize. Further studies should investigate the sources of these predictions more thoroughly. For example, it could be important whether or not the research hypothesis was perceived as plausible. Although we have not covered this topic in the current studies, we assume that both research hypotheses (temperature influences perception of social proximity, physical inhibition transfers to cognitive processes) are plausible. Plausibility will surely affect estimations of effect size, beyond significance. Sample size may be less influenced by considerations of plausibility, however.

Estimating statistical values obviously was a very difficult task for participants. Note that we used students in their statistics course as participants, and we incentivized the task. Nevertheless, a good share of our participants was unwilling to provide plausible estimates. We excluded those from our analysis. However, the remaining participants had also difficulties estimating some values, for instance means, and, most notably, standard deviations. This difficulty was most evident in the locomotion study, where the estimates were far off. As it seems, estimating such values is not part of their training in statistics. In conjunction with a difficult dependent variable like reaction time measured in milliseconds, this may render such a task really difficult. However, getting a grasp on what sample statistics mean, and what their plausible range can be, is important for a thorough understanding of statistical results. Therefore, tasks like ours can be used not only for investigating statistical intuitions, but also for providing training in these intuitions.

## Conclusions

This study showed that students have a limited understanding of the underlying concepts of statistical inference. Statistical and practical significance were not distinguished properly. Since some of these students might be future researchers this lack of understanding can have a colossal impact on the whole research field [66]. Indeed, recent analysis of effect size reporting practices found that discrepancies between statistical and practical significances were rarely discussed by the authors of articles [67,68]. But as Kline [16] pointed out, circulating misconceptions like the magnitude fallacy may not be solely the fault of users; rather the logical foundation of contemporary NHST is not entirely consistent. To prevent future confusion about statistical and practical significance effect sizes should be routinely reported as recommended by the major associations in psychology [69] and education [70]. The publication manual ([69], p. 34) states: ‘For the reader to appreciate the magnitude or importance of a study’s finding, it is almost always necessary to include some measure of effect size in the results section.’ But as Henson [71] clarified, a realisation thereof will need continued education and explication. Our findings testify to this conclusion.

Our students tend to interpret the label ‘significant’ as showing that a study found a nontrivial effect size, rather than that it was large. This is legitimate, and therefore not a fallacy proper. However, in many cases significance is achieved through questionable research practices, among which adaptive sampling (i.e., increasing sample size to achieve significance) is a prominent one [72]. In addition, there is a significant correlation between sample size and effect size in psychological research [73], indicating that significance is often due to large samples, rather than to large effects. Our findings thus show that students still believe in the seriousness of scientific conduct, and that scientific journals are filled with papers that have substance in a practical sense: we must not jeopardize this positive view.

## Appendix: Task description and instruction

Dear participant, thank you for taking part in our survey. You will see descriptions of two scientific research papers and we ask you to indicate your personal guess on several features of these studies (sample size, p-value, …). It is important that you give your personal and intuitive estimates.

You must not be shy in delivering your estimates, even if you are not sure at all. We are aware that this may be a difficult task for you – yet, please try.

### Task 1: The influence of warmth on social distance

In this study researchers investigated the influence of warmth on social distance. The hypothesis was that warmth leads to social closeness. There were two groups to investigate this hypothesis:

Participants of group 1 held a warm drink in their hand before filling in a questionnaire. Participants of group 2 held a cold drink in their hands before they filled in the same questionnaire. Participants were told to think about a known person and had to estimate their felt closeness to this person. They had to indicate closeness on a scale from 1–7, whereas 1 means ‘very close’ and 7 means ‘very distant’.

The closeness ratings of the participants of group 1 were then compared to the closeness ratings of group 2.

Researchers found a statistically significant [non-significant] effect in this study.

### Task 2: The influence of body movement on information processing speed

Previous studies have shown that body movements can influence cognitive processes. For instance, it has been shown that movements like bending an arm for pulling an object nearer go along with diminished cognitive control. Likewise, participants showed more cognitive control during movements pushing away from the body. In this study, the influence of movement of the complete body (stepping forward vs. stepping backward) on speed of information processing was investigated.

The hypothesis was that stepping back leads to more cognitive control, i.e., more capacity. There were two conditions in this study: In the first condition participants were taking four steps forwards, and in the second condition participants were taking four steps backwards. Directly afterwards they worked on a test capturing attention in which their responses were measured in milliseconds. The mean reaction time of the stepping forward-condition was compared to the mean reaction time of the stepping backward-condition.

*Researchers found a statistically significant [non-significant] effect in this study.*
